# Inclusion of Pork Meat in the Diets of Young Women Reduces Their Intakes of Energy-Dense, Nutrient-Poor Foods: Results from a Randomized Controlled Tria

**DOI:** 10.3390/nu6062320

**Published:** 2014-06-19

**Authors:** Jennifer O. McArthur, Natalie M. Gough, Peter Petocz, Samir Samman

**Affiliations:** 1 Discipline of Nutrition and Metabolism, School of Molecular Bioscience, The University of Sydney, NSW 2006, Australia; E-Mails: jennifer.mcarthur@sydney.edu.au (J.O.M.); ngou3547@uni.sydney.edu.au (N.M.G.); 2 Department of Statistics, Macquarie University, North Ryde, NSW 2109, Australia; E-Mail: peter.petocz@mq.edu.au

**Keywords:** diet, pork, food groups, food habits, young women

## Abstract

Adherence of young women to dietary recommendations has been examined predominantly by surveys. This study aimed to determine the quality of women’s diets relative to the Australian Guide to Healthy Eating (AGHE); and to evaluate dietary changes during an intervention trial with pork meat or an iron supplement. A 12-week randomized trial was conducted in young women who were assigned to one of three groups. They maintained three, seven-day food diaries while continuing their routine diet (CG); taking an iron supplement (SG); or incorporating into their diets 500 g/week of pork (PG). Participants (*n* = 58) provided dietary information on 1218 diary-days. The serves consumed from the *vegetable*, *fruit* and *dairy* groups were lower (*p* < 0.001), and from the *meat and alternatives* group greater (*p* < 0.001) than the recommended serves. PG consumed significantly fewer (*p* < 0.001) serves of “*extra*” foods, and ate *fruit* more frequently (*p* < 0.001) than CG and SG. The participants’ dietary self-assessment showed poor agreement with the AGHE description of “serve”. The inclusion of pork in the diets of young women is associated with the reduced consumption of energy-dense nutrient-poor “*extra*” foods and increased frequency of fruit intake. The effect may be explained by diverse factors such as increased food knowledge, cooking skills and the effect of pork on satiety.

## 1. Introduction

Compromised iron status persists in young women despite access to an adequate food supply [1]. This raises questions for nutrition experts who evaluate and disseminate knowledge about food in order to help individuals establish healthy eating practices. As part of a three-month trial aimed at improving iron status in women by increasing their intakes of pork meat or iron supplements, we assessed the women’s daily food intake in order to study their food and nutrient management practices [2,3]. Analysis of food frequency questionnaires during the trial revealed that the intakes of several micronutrients were below the Estimated Average Requirement [2,3]. A closer examination is undertaken in the present study of the women’s customary diets and the impact of the intervention in relation to meeting the Dietary Guidelines for Australians (DGA). The DGA are publicly available along with complementary resources known as the Australian Guide to Healthy Eating (AGHE) [4]. These classify foods into six groups: five core food groups, according to their nutrient profile, and one group (“*extra*”) consisting of foods that are energy-dense and nutrient-poor.

The Food and Agriculture Organization reviewed the effectiveness of nutrition education and the promotion of healthy diets that were consistent with food based dietary guidelines. It is noted that there have been numerous “eat more” and “eat less” campaigns since 1992. Nevertheless, public awareness nutrition campaigns have focused predominantly on fruit and vegetable intakes [5]. They have helped to increase the intakes of fruit and vegetables in the early 1990s [6,7]. However, Casagrande *et al.* reported that by 2002 intakes had fallen to levels consumed in 1995 [8]. Ammerman *et al.* reported significant increases in the intakes of fruit and vegetables that were sustained; however, these remained lower than the national recommendations [9]. Ball *et al.* [10] studied middle-aged Australian women finding they did not meet the guidelines. Pollard *et al.* [11] reported positive changes in knowledge and behaviors following the “Go for 2 [fruit] & 5 [vegetables]” campaign. Nevertheless, it was noted this did not translate to an increased number of fruit and vegetable servings, either due to perceptions held that current intakes were adequate, or because individuals considered themselves too time poor for the preparation of vegetables [11].

National data aimed at determining nutrient intakes and understanding of dietary guidelines are derived mainly from 24 h dietary recalls, and are cross-sectional in nature [12,13,14,15,16,17]. Reports of dietary analyses continue to highlight the nutrients at risk for each stage of the lifecycle, including for women of reproductive age [8,18,19]. However, the adherence of young women to the food based dietary recommendations has been poorly studied, and the effectiveness of the educational tools for this sector of the population has been limited mainly to surveys, with and without 24 h recalls or 3-day food diaries [15,17,20,21]. Therefore, an opportunity exists in the present study in young women: (i) to ascertain the habitual food intakes derived from 21 days of food diaries (FD); (ii) to gauge the quality of their diets relative to the AGHE; and (iii) to evaluate changes in food intake during their participation in a dietary intervention trial that involved the consumption of pork meat or an iron supplement.

## 2. Experimental Section

### 2.1. Participants and Trial Design

Women were recruited through advertising and leaflet distribution on the University of Sydney campus. Potential volunteers were screened by using a short questionnaire and selected based on age (18–35 years). Exclusion criteria included vegetarians, those who were pregnant, lactating, or reported a major illness (e.g., gastrointestinal disease), and those consuming nutritional supplements or medication. The University of Sydney Human Ethics Review Committee approved the study and all subjects gave written informed consent prior to their participation.

The trial design and biomarker outcome measures are described elsewhere [2]. Briefly, the 12-week study had a parallel design with subjects randomly assigned, by using computer-generated random numbers, to one of 3 groups: control, pork meat diet intervention or supplement intervention. As noted previously [2], a sample of 63 women was required based on an anticipated increase in serum ferritin concentrations (a difference of 50% of the average baseline value). Subjects who were assigned to the control group (CG) were asked to maintain their existing eating patterns and were provided with general dietary advice. They also consumed a placebo capsule that contained 220 mg cellulose. Participants allocated to the pork diet group (PG) were asked to incorporate a minimum of 500 g of pork meat (3–4 serves) into their meals each week without reducing their current intake of red meat. They were given free choice as to serving size, time of consumption or method of preparation. Weighed and frozen portions of pork meat were provided every two weeks, and the participants’ intake of pork meat since last collection was recorded. The meat was provided to the volunteers as part of their participation in the study, and they were asked also to consume a placebo capsule. Participants in the iron supplement group (SG) were provided with general dietary advice, but were given daily low-dose iron supplements that contained 37.4 mg elemental iron in the form of ferrous gluconate. The scheduling of dietetic consultations during the study prevented discussions between the volunteers; however, it was not possible to blind participants randomized to PG.

### 2.2. Data Collection and Analysis

Food intake data were sourced on three separate occasions (FD1, FD2 and FD3) from 7-day food diaries that were maintained by the participants who recorded all foods and drinks consumed during weeks 4, 8 and 12 of the study [22]. The participants recorded their food intakes for a total of 21 days onto sequentially numbered recording sheets that were checked and entries clarified by the dietitian during the monthly consultations [23]. Participants were asked also to record, on a second sheet, the number of serves that they had consumed from each of the AGHE food groups [4]. Following the collection of each diary, the researchers would independently calculate and record the daily food group count for each participant. Comparisons were made between the participant and researcher count and differences noted. Baseline dietary data using validated food frequency questionnaires (FFQ) [24] were collected at weeks 0 and 12 and intake comparisons were made between study groups.

Intakes of the various food groups over the course of the study were compared using ANOVA. Chi-square tests were used to determine the association between recorded intakes and the AGHE recommendations, and whether the intervention groups (CG, PG and SG) were significantly different from each other. Student’s *t*-tests were used to determine whether the number of serves consumed by each group of participants was statistically different from the AGHE recommendations. A value of *p* < 0.01 was taken to designate statistical significance, with 0.01 ≤ *p* ≤ 0.05 indicating marginal significance. We took a conservative approach and interpreted *p* < 0.05 as statistically marginal due to the large number of tests results under investigation. SPSS v20 was used for all statistical calculations.

## 3. Results

There were 65 participants with 58 providing complete dietary data (CG *n* = 20; PG *n* = 16; SG *n* = 22). Each participant recorded data on 21 days thus providing a total of 1218 diary-days for analysis (CG = 420 records; PG = 336 records; and SG = 462 records). The participants were 24.4 ± 4.2 years at study commencement, and within the healthy weight range throughout the study. The participants were university students, not living in university colleges; and were of European (66%) or Asian (34%) descent. The mean intakes for the three 7-day diary collection periods are shown in Table 1.

**Table 1:** Number of serves ^1^ consumed by participants in the control (CG), pork diet (PG) and supplement (SG) groups during each collection period.

Food Group (Recommended Serves)	Week ^8^	CG	PG	SG
***Grain*^2^ (4 serves)**	4	3.29 ± 1.08	3.06 ± 0.52	3.80 ± 1.06
	8	3.43 ± 1.18	3.09 ± 0.71	3.34 ± 1.04
	12	3.48 ± 0.91	3.00 ± 0.92	3.35 ± 1.49
***Vegetable*^3^ (5 serves)**	4	2.31 ± 0.66	2.22 ± 0.65	2.29 ± 0.96
	8	2.21 ± 0.80	2.32 ± 0.90	2.13 ± 1.10
	12	2.04 ± 0.83	2.58 ± 0.69	1.87 ± 1.06
***Fruit*^4^ (2 serves)**	4	1.03 ± 0.58	1.35 ± 0.47	1.29 ± 0.63
	8	0.95 ± 0.55	1.32 ± 0.64	1.08 ± 0.68
	12	0.83 ± 0.55	1.40 ± 0.63	1.13 ± 0.87
***Dairy*^5^ (2 serves)**	4	0.83 ± 0.47	0.74 ± 0.47	0.76 ± 0.52
	8	0.90 ± 0.49	0.74 ± 0.47	0.66 ± 0.51
	12	0.99 ± 0.45	0.71 ± 0.47	0.66 ± 0.49
***Meat and alternatives*^6^ (1 serve)**	4	1.61 ± 0.51	1.69 ± 0.35	1.66 ± 0.56
	8	1.63 ± 0.50	1.77 ± 0.33	1.64 ± 0.51
	12	1.74 ± 0.69	1.79 ± 0.35	1.65 ± 0.69
***Extra*^7^ (max 2.5 serves)**	4	2.64 ± 1.71	1.81 ± 0.83	2.24 ± 1.31
	8	2.49 ± 1.57	1.37 ± 0.96	4.07 ± 6.77
	12	2.18 ± 1.26	1.27 ± 0.50	2.17 ± 1.03

^1^ Values are mean ± standard deviation; ^2^ bread, cereals, rice, pasta, and noodles; ^3^ vegetables and legumes; ^4^ fresh, canned and dried fruit; ^5^ milk, cheese, yoghurt and alternatives; ^6^ meat, fish, poultry, eggs, legumes and nuts; ^7^ 600 kJ from items such as, doughnuts, biscuits, cakes, chocolate, alcoholic drinks, soft drinks, meat pies, hot chips; ^8^ Each week represents 7 days of dietary data.

As reported previously [2] there were no significant differences in intakes for each of the food groups across the collection periods for all intervention groups (Table 2). The data were therefore pooled for each of the intervention groups. Analysis of FFQ revealed no significant difference in macronutrients between the intervention groups at week 0 and no significant difference in energy intakes between week 0 and week 12 [2].

**Table 2:** Protein, fat and carbohydrate intake (% energy) in the control (CG), pork diet (PG) and iron supplement (SG) groups at week 0.

Nutrient	Week 0		
	CG ^a^	PG ^a^	SG ^a^
Protein (% energy)	21.0 ± 5.0	19.6 ± 3.1	19.9 ± 3.3
Carbohydrate (% energy)	42.4 ± 7.4	44.0 ± 5.4	46.6 ± 6.5
Fat (% energy)	32.9 ± 6.2	34.4 ± 5.9	31.8 ± 6.2

^a^ Values are mean ± standard deviation. CG *n* = 20, PG *n* = 16, SG *n* = 22.

**Figure 1:**
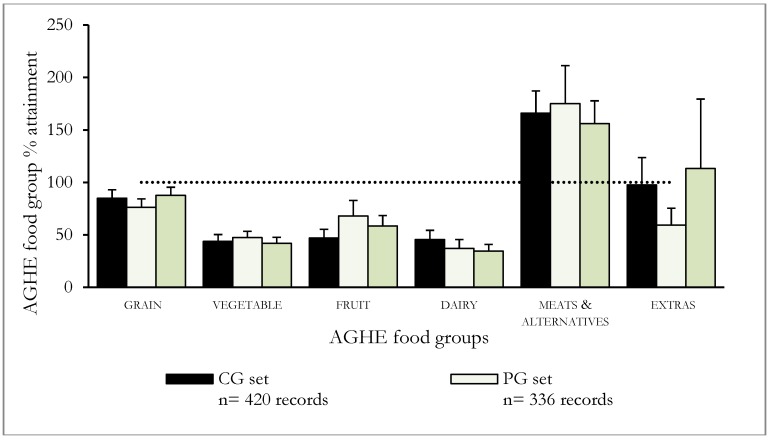
Consumption of Australian Guide to Healthy Eating (AGHE) food groups ^1^ expressed as percentage of recommendations by control (CG), pork diet (PG) and iron supplement (SG) groups. Mean of 21 days of food diary recorded over a 12-week intervention period.

^1^ GRAIN: bread, cereals, rice, pasta, and noodles; VEGETABLE: vegetables and legumes; FRUIT: fresh, canned and dried fruit; DAIRY: milk, cheese, yoghurt and alternatives; MEATS & ALTERNATIVES: meat, fish, poultry, eggs, legumes and nuts; EXTRAS: 600 kJ from items such as, doughnuts, biscuits, cakes, chocolate, alcoholic drinks, soft drinks, meat pies, hot chips.

Figure 1 shows the number of serves consumed by the participants expressed as a percentage of AGHE and the results of statistical comparisons with recommendations (from *t-*tests) are shown in Table 3. Compared to the AGHE recommendations, the number of serves consumed from the *vegetable*, *fruit* and *dairy* groups by all study groups were significantly lower (*p* < 0.001), and from the *meat and alternatives* group significantly greater (*p* < 0.001) than the recommended number of serves. The intervention with pork meat had an effect on the intake of “*extra*” foods: PG consumed significantly less (59%; *p* < 0.001) than the recommended maximum number of serves of “extra” foods, unlike CG and SG.

**Table 3:** Mean number of serves ^1^ consumed by the control (CG), pork diet (PG) and supplement (SG) groups.

Food Group (Recommended Serves)	Study Set	Mean	SD	*p* value ^2^
***Grain*^3^ (4 serves)**	CG	3.40	0.95	0.011
	PG	3.05	0.58	<0.001
	SG	3.51	0.97	0.026
***Vegetable*^4^ (5 serves)**	CG	2.19	0.57	<0.001
	PG	2.34	0.69	<0.001
	SG	2.10	0.89	<0.001
***Fruit*^5^ (2 serves)**	CG	0.94	0.49	<0.001
	PG	1.36	0.49	<0.001
	SG	1.67	0.70	<0.001
***Dairy*^6^ (2 serves)**	CG	0.91	0.40	<0.001
	PG	0.75	0.42	<0.001
	SG	0.69	0.41	<0.001
***Meat and alternatives*^7^ (1 serve)**	CG	1.66	0.47	<0.001
	PG	1.75	0.24	<0.001
	SG	1.56	0.52	<0.001
***Extra*^8^ (max 2.5 serves)**	CG	2.44	1.41	0.845
	PG	1.48	0.54	<0.001
	SG	2.82	2.46	0.541

^1^ Values are the means obtained from 21 days of dietary data; ^2^ *p* value from student *t*-tests to compare against number of recommended serves; ^3^ bread, cereals, rice, pasta, and noodles; ^4^ vegetables and legumes; ^5^ fresh, canned and dried fruit; ^6^ milk, cheese, yoghurt and alternatives; ^7^ meat, fish, poultry, eggs, legumes and nuts; ^8^ 600 kJ from items such as, doughnuts, biscuits, cakes, chocolate, alcoholic drinks, soft drinks, meat pies, hot chips.

The percentage of days during the collection period that the AGHE recommendations were achieved is shown in Table 4. All groups consumed less than the recommended number of serves for *grain*, *vegetable* and *dairy*, with no differences in frequency of intake between groups. None of the participants met the *grain* recommendation for more than 8 of the 21 FD days (data not shown). No associations were found between study groups and serves of *grain* consumed each day.

All the intervention groups consumed significantly less *fruit* than the recommended number of servings (Figure 1); however, significantly different patterns of association were found between the study sets (*p* < 0.001; Table 4). Examination of the frequencies showed that PG consumed *fruit* commensurate with or greater than the recommendations approximately 40% of the time (*i.e.*, 135 of 336 days) while this was lower for CG (24%) and SG (31%).

All groups consumed above the recommended number of serves from the *meat and alternatives* food group (*p* < 0.001, Figure 1). Participants in PG, who were directed to consume pork meat as a component of the trial intervention, increased their intake of red meat, while reducing their intake of poultry and nuts. Significant (*p* < 0.001) associations were observed between the groups and the percentage of days that the recommended number of serves was consumed (*p* < 0.001) (Table 4). PG omitted *meat and alternatives* on a fewer number days (2.5%) compared to CG and SG who failed to consume foods from this food group on 14.9% and 12.4% of the study days, respectively. Unlike participants in CG (25%) and SG (32%), all participants in PG met the secondary AGHE recommendation of consuming 3–4 serves of red meat each week for the duration of the collection period of 21 days.

**Table 4:** Percentage of days during the 21-day collection period that the AGHE food group recommendations were consumed by the control (CG), pork diet (PG) and supplement (SG) groups.

Food Group (Recommended Serves)	Group	Percentage Days (%)				*p* value ^1^
		Not Consumed	<AGHE	=AGHE ^a^	>AGHE ^a^	
***Grain*^2^ (4 serves)**	CG	2.6	56.7	40.7	0.0	ns
	PG	1.5	62.8	35.7	0.0	
	SG	2.1	62.7	35.2	0.0	
***Vegetable*^3^ (5 serves)**	CG	11.8	82.9	2.6	2.6	ns
	PG	10.1	82.4	3.5	4.0	
	SG	13.8	79.3	3.4	3.5	
***Fruit*^4^ (2 serves)**	CG	35.6	40.4	15.6	8.4	<0.001
	PG	19.6	40.2	22.6	17.6	
	SG	28.7	40.1	16.9	14.3	
**Dairy ^5^ (2 serves)**	CG	24.0	62.3	8.2	5.5	ns
	PG	32.7	57.2	6.1	4.0	
	SG	34.9	55.4	5.8	3.9	
***Meat and alternatives*^6^ (1 serve)**	CG	14.9	8.2	13.9	63.0	<0.001
	PG	2.5	5.5	18.1	73.9	
	SG	12.4	8.9	21.1	57.6	
***Extra*^7^ (max 2.5 serves)**	CG	24.8	52.6	4.1	18.5	<0.001
	PG	32.2	53.8	1.5	12.5	
	SG	21.3	51.7	3.0	24.0	

^1^ *p* value from Chi-square tests conducted on the frequency distributions; ^2^ bread, cereals, rice, pasta, and noodles; ^3^ vegetables and legumes; ^4^ fresh, canned and dried fruit; ^5^ milk, cheese, yoghurt and alternatives; ^6^ meat, fish, poultry, eggs, legumes and nuts; ^7^ 600 kJ from items such as, doughnuts, biscuits, cakes, chocolate, alcoholic drinks, soft drinks, meat pies, hot chips.

Participants in PG exceeded the recommendation for “*extra*” foods on fewer days (12.5%) compared to CG (18.5%) and SG (24%). There were significant (*p* < 0.001) associations observed between the intervention groups and the number of serves of “*extra*” foods that were consumed as well as the percentage of days that the serves were consumed.

Lunch meals consumed that were prepared outside the home (restaurant or take-away) were similar for all groups (40%); however, overall PG consumed less externally prepared meals—breakfast 17% (PG) compared to 22% (CG) and 23% (SG); and dinner, 26% (PG) and 49% both for CG and SG.

The participants’ self-assessments of the number of serves they consumed each day were compared to the AGHE description of “serve” for each food group. Agreement ranged from 20% (*vegetable*) to 60% (*fruit*). The vegetables that the participants miscalculated most often were broccoli, corn, peas and sweet potato. The higher agreement with the *fruit* group was aided by the fact that most fruits have one piece of the whole fruit (e.g., one apple) equaling one AGHE serve. The meals that were the most difficult for the participants to calculate were mixed dishes such as stir-fry and mixed pasta.

## 4. Discussion

In the present study young women recorded their dietary intakes for 21 days as part of an intervention trial lasting three months. None of the participants achieved the recommended number of serves from the *grain*, *vegetable*, *fruit*, or *dairy* (*p* < 0.001) food groups, and despite exceeding the *meat and alternatives* recommendation, only those who were assigned to PG consumed the secondary recommendation of 3–4 serves of red meat per week. PG consumed significantly fewer energy-dense nutrient-poor “*extra*” foods, and ate fruit more frequently than subjects in CG and SG.

Kolodinsky *et al.* [25] reported that college students with knowledge of dietary guidelines were better equipped to meet the recommended intakes of fruit, dairy and grain-based foods; and Anding *et al.* [20] reported that the majority of the female students were meeting at least one of the seven (American) dietary guidelines. Georgeou *et al.* [26] report that diet quality is higher for undergraduate students than graduates and their working peers. In the 20–29 year age category in Australia, 33% attend tertiary institutions [27] and more than half are female [28]. These statistics suggest that a large proportion of young women are consuming diets of poor nutritional quality. These and other studies that report on consumer understanding are limited by their study design in their ability to report accurately whether the participants were following the dietary guidelines [29]. In the present study we determined the participants’ food group knowledge using a less intense questioning protocol than Kolodinsky *et al.* [25], and we ascertained that a large percentage of participants could recall the AGHE serving recommendations. Despite their knowledge of the AGHE, there was limited translation of knowledge into action, as evidenced from dietary data that were collected over 21 days.

We showed previously in young women that the avoidance of animal foods is reported in 23% of participants, and resulted in lower intakes of omega-3 fatty acids, vitamin B_12_, selenium and zinc compared to non-avoidance [30]. A high percentage of the young women considered themselves overweight or obese, despite having a BMI in the normal range. Assessment of their eating behavior showed that 27% were classified in the high-restraint category, that is, individuals who avoid specific foods in order to maintain their body-weight [31]. Iron intake from animal sources was significantly lower with the increase in avoidance of meat and poultry [30]. The present findings show that the recommendation for young women to consume one serving from the *meat and alternatives* group daily was exceeded. This food category is heterogeneous [4], containing a range of plant and animal foods that are high in protein (the intention of this recommendation). However, many foods within this category are poor sources of bioavailable iron and some, such as legumes and nuts, contain inhibitors of iron absorption. The incorporation of pork meat in PG was accommodated into the participants’ diets by decreasing the intake of poultry and nuts, thereby increasing the intake of haeme iron.

Henson *et al.* [32] investigated the difficulty that individuals encounter when incorporating the changes associated with healthier eating, and determined that increasing fruit and vegetable intakes was the most challenging. Exploring the eating practices of young women in the current study, while analyzing the intakes during the dietary intervention, enabled insight concerning dietary change. The inclusion of pork meat in the present study impacted favorably on the ability of the participants to meet the recommended number of serves of *fruit* and “*extra*” foods. PG consumed significantly less energy-dense nutrient-poor food items than CG and SG. Foods from the “*extra*” foods group that were avoided by PG included toasted, flavored breads, such as banana-bread, confectionery and chocolate-based snack foods. These items were replaced with fresh fruit—e.g., bananas or kiwifruit. The food diaries indicate a larger percentage of nutritionally complete meals were ingested by PG, and accordingly satiation may account for fewer energy-dense mid-meals. This apparent favorable exchange was reflected in the PG participants’ dietary records such that the frequency of fruit consumption increased, and the number of days that the participants achieved the recommendation for the number of serves of *fruit* also increased.

The consumption of energy-dense, nutrient-poor foods is of public health concern. These foods contribute few micronutrients to the diet, but contain substantial amounts of fat, sugar or both, and are high in energy. Patterns of consumption of such foods may contribute to excessive energy intakes and development of chronic disease [33]. In the diets of Australian adults, “*extra*” foods such as fried potatoes, cakes, muffins, soft drinks and confectionery contribute up to 36% to the daily energy intake [34]. Similar data are available for American adults who consume approximately 31% of their energy intake from nutrient-poor energy-dense foods. It has been estimated that the likelihood of meeting the recommended dietary allowance for protein and selected micronutrients, decreased with increasing intakes of items such as those categorized as “*extra*” foods [35]. A sustained low intake of micronutrients, together with a propensity for meat avoidance, may lead to adverse health effects in the longer-term, such as compromised immune function and iron deficiency anemia.

In a longitudinal study of weight change in Australian women in their 20s, fewer than half of the women who were surveyed were able to maintain their weight over a 4 years period. The authors suggest that during early adulthood in women an opportunity exists for encouraging physical activity and promoting fewer take-away foods, in order to maintain a healthy weight [36]. In the present study, the necessity for participants to construct meals around the pork meat, which was provided to them as fresh (uncooked) cuts, appears to have contributed to an improved diet possibly because the additional 3–4 pork-containing meals each week replaced take-away meals, and of necessity, increased the participants’ cooking skills and food knowledge that are required to prepare a meal. We considered the provision of pork meat enabled student monies to be re-directed to enable consumption of a higher quality diet. However, this is unlikely as the cost to students of purchasing 2–3 take-away meals per week was greater than the cost of preparing 3–4 pork meat meals when all the fresh ingredients were costed. Student funds do not appear to be the driving force for food selection, and Rao *et al.* [37] determined that the cost of a healthy diet over a poor diet to be less than $2 per week—an affordable alternative for the control and supplement groups.

Recent studies investigating the influences on the intake of snack foods, which are a major constituent of the “*extra*” foods classification, have focused on environmental cues, container size, and satiety [38,39,40]. Charlton *et al.* [41] investigated the effect of pork consumption on appetite and satiety. The authors found that after consumption of test meals that contained either pork, beef or chicken, significantly higher concentrations of the satiety hormone peptide YY were observed in those who consumed the pork meal as compared to chicken. The observation in the present study that the consumption of pork is associated with fewer “*extra*” foods suggests that the inclusion of pork meat may have contributed to satiety and therefore elicited a favorable change in eating behavior.

The use of pork meat and the requirement to swallow daily capsules precluded some individuals from participating and the poor knowledge of food and cooking skills restricted the choices of meat cuts that could be used in the intervention.

## 5. Conclusions

The participants in the present study were unable to achieve the recommended number of serves for *grain*, *vegetable*, *fruit* or *dairy*. Intakes from the *meat and alternatives* group exceeded the recommendations although the foods consumed were low in iron or consisted of foods that are poor sources of bioavailable iron, such as nuts. The incorporation of pork meat into the diet was associated with an improved nutritional profile, indicated by a decrease in the consumption of energy-dense nutrient-poor snacks (“*extra*” foods), and an increase in the frequency of *fruit* consumption. We postulate that the mechanism for this change is related to diverse factors such as the effect of pork consumption on satiety, food knowledge and the necessity to prepare complete meals.
